# Syzygium Cordatum Hochst. ex Krauss: An Overview of Its Ethnobotany, Phytochemistry and Pharmacological Properties

**DOI:** 10.3390/molecules23051084

**Published:** 2018-05-04

**Authors:** Alfred Maroyi

**Affiliations:** Medicinal Plants and Economic Development (MPED) Research Centre, Department of Botany, University of Fort Hare, Private Bag X1314, Alice 5700, South Africa; amaroyi@ufh.ac.za; Tel.: +002-77-1960-0326

**Keywords:** ethnopharmacological, Myrtaceae, phytochemistry, *Syzygium cordatum*, tropical Africa

## Abstract

*Syzygium cordatum* is a valuable medicinal plant in the materia medica of east and southern Africa. The aim of this study was to review the botany, medicinal uses, phytochemistry and ethnopharmacological properties of *S. cordatum*. Relevant literature search was carried out using internet sources such as ACS, Web of Science, Wiley, SpringerLink, Scopus, Mendeley, Google Scholar, Pubmed, SciFinder, BioMed Central, Science Direct and Elsevier. Other literature sources were conference papers, book chapters, books, theses and websites. The leaves, roots, bark and fruits of *S. cordatum* are used as ethnomedicines against 24 human diseases such as gastro-intestinal disorders, burns, sores, wounds, colds, cough, respiratory complaints, sexually transmitted infections (STIs), tuberculosis, fever and malaria. Several phytochemical compounds including alkaloids, anthocyanidin, essential oils, flavonoids, leucoanthocyanidin, phenols, phytosterols, saponins, simple sugars, terpenoids and triterpenoid have been identified from *S. cordatum*. Pharmacological evaluations revealed that *S. cordatum* is characterized by several biological activities including antibacterial, antifungal, antidiarrheal, anti-sexually transmitted infections, antidiabetic, anticholinesterase, anti-inflammatory, antileishmanial, antioxidant, antiplasmodial and anti-proteus. These pharmacological findings lend credence to the traditional ethnomedicinal uses and ethnopharmacological importance of *S. cordatum*. Future research on the species should identify the biological compounds, their mode of action and physiological pathways and clinical relevance.

## 1. Introduction

*Syzygium cordatum* Hochst. ex Krauss (family Myrtaceae) is a valuable herbal medicine in east and southern Africa and it is included in the monographic guide of the most valuable herbal medicines in South Africa [1]. In Uganda, a survey conducted by Katumba et al. [2] aimed at identifying medicinal plant species that are widely used and traded in the country identified *S. cordatum* as one of the priority species for domestication and on-farm planting to promote sustainable utilization of the species. In Swaziland, *S. cordatum* is regarded as a multipurpose plant species which is important for local livelihoods as herbal medicine, food source as its fruits are edible, source of fuel wood and charcoal, timber, building materials and fences, and for landscaping purposes as an ornamental plant [3]. Similarly, in South Africa, *S. cordatum* is used as an ornamental plant; it is an important source of strong and durable timber; the fruits are consumed by humans and animals; the fruits are made into potent alcoholic drink; the bark and leaves are consumed by livestock and game; and the bark and fruits are used for dyeing [4,5,6]. According to van Wyk [7], *S. cordatum* bark has commercial potential as remedy for respiratory ailments and stomach complaints. Research focusing on African medicinal and aromatic plants of commercial importance revealed that the bark of *S. cordatum* feature prominently in Zimbabwe, South Africa and Kenya as traditional medicine for diarrhea and stomach ailments [7]. It is within this background that the ethnobotany, phytochemistry and pharmacological properties of *S. cordatum* are reviewed. The collection and utilization of *S. cordatum* as herbal medicine in east and southern Africa attracted a lot of interest over the years as demonstrated by ethnopharmacological research focusing on the species [1,2,7,8,9]. The current study is therefore, aimed at reviewing the ethnobotany, phytochemistry and pharmacological properties of *S. cordatum* throughout its distributional range. It is hoped that this information will identify baseline data required for future research focusing on the ethnopharmacology of the species.

## 2. Botanical Profile and Taxonomy of *S. cordatum*

*Syzygium cordatum* belongs to the Myrtaceae or myrtle family. The Myrtaceae family is a made up of about 133 genera and in excess of 3800 species with centers of diversity of the family in Australia, tropical to southern temperate America and southeast Asia [10]. *Syzygium* Gaertn. genus is the largest woody genus not only of the family Myrtaceae but of the flowering plants in the world, characterized by 1200–1800 species distributed throughout tropics and subtropics in Africa, Asia and Australia [11]. In mainland Africa, the genus is represented by 35 taxa while the western Indian Ocean islands and the Mascarenes are represented by 35 and 22 taxa, respectively [11]. The generic name “*Syzygium”* is based on a Latin word “syzygia” and Greek word “syzygos” meaning yorked, coupled or partnered, perhaps referring to paired branches and leaves of the species [12,13]. The specific name “*cordatum*” is derived from a Latin term “cordatus” in reference to heart-shaped or cordate, as the base of the leaves of the species is heart-shaped [12]. Most of the common names such as water berry, water tree and water wood indicate that the species often grows near water [4,12]. Two synonyms, that is, *Eugenia cordata* (Hochst. ex Krauss) G. Lawson and *S. cordatum* var. *gracile* Amshoff, are associated with *S. cordatum* [13].

*Syzygium cordatum* is a small-sized tree or large shrub that is evergreen, reaching about 18 m in height and the bole can grow up to 60 cm in diameter [12,14]. The bole is seldom straight but often branched, gnarled and at times buttressed. When young, the trunks are banded and blotched in grey and white and are fairly smooth. In old trees, the bark is dark and light grey or reddish, thick and fissured, and can be pulled off in thick and cork-like pieces [12]. The leaves are opposite, simple, entire with waxed margins, blue-green in above, paler green below, thick, leathery and smooth. The blade varies in shape from oblong to almost round, base cordate, clasping the stem with rounded or bluntly pointed tips. The midrib, lateral and net veins are conspicuous. The inflorescence is a terminal cyme with many-flowers. The flowers are bisexual, regular, white, pinkish or yellowish in color. The fruits are oval berries, red to dark-purple when ripe [14].

*Syzygium cordatum* is found growing close to water, along streams and rivers or in damp areas in swamps, on forest margins, in open grasslands, among rocks and also on roadside banks in higher rainfall areas [13]. *Syzygium cordatum* is known to occur in Angola, Burundi, the Democratic Republic of Congo, Gabon, Kenya, Tanzania, Malawi, Botswana, Mozambique, Zimbabwe, Namibia, Swaziland, South Africa, Uganda and Zambia at altitude ranging 50–2300 m above sea level [13].

## 3. Medicinal Uses

**Figure 1:**
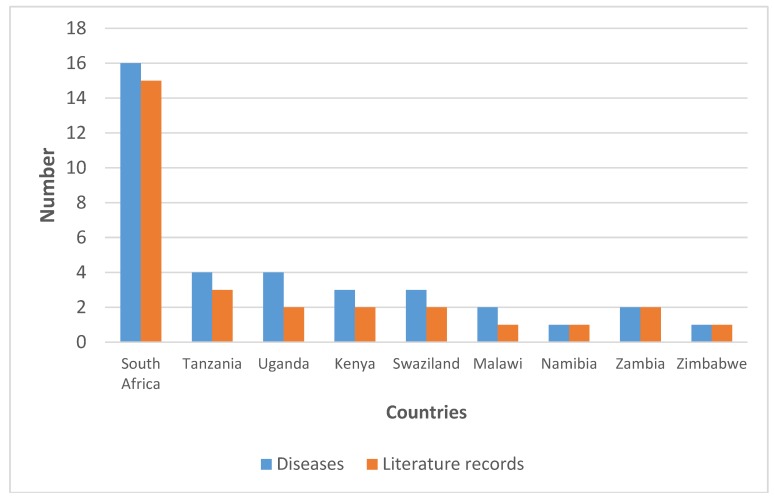
Diseases and ailments treated by *Syzygium cordatum* in east and southern Africa.

The bark, fruits, leaves and roots of *S. cordatum* are used to cure at least 24 human diseases in east and southern Africa (Table 1). Ethnomedicinal information has been found in Kenya, Malawi, Namibia, South Africa, Swaziland, Tanzania, Uganda, Zambia and Zimbabwe, representing 60% of the countries where *S. cordatum* is native. South Africa has the highest number of medicinal uses with 16 records of human diseases treated or managed by concoctions prepared from *S. cordatum*, based on 15 literature records (Figure 1). Tanzania has four medicinal uses recorded in three sources, followed by Uganda with four uses based on two sources, and Kenya and Swaziland with three uses each based on two literature records (Figure 1). Gastro-intestinal disorders such as diarrhea, dysentery and stomach problems; burns, sores and wounds; colds, cough and respiratory complaints; sexually transmitted infections (STIs); tuberculosis (TB); fever; and malaria (Table 2) are the most commonly treated human diseases and ailments using concoctions prepared from *S. cordatum.*

In traditional medicine, stem bark and root infusion of *S. cordatum* is used against diarrhea in Kenya, Zambia, Malawi, South Africa and Namibia [1,5,15,16,17,18,19,20] (Table 1). Bark or leaf decoction of *S. cordatum* is used against dysentery in Malawi [15] and gastro-intestinal complications in Kenya [21]. In Swaziland, stem bark of *S. cordatum* is mixed with bark of *Breonadia salicina* (Vahl) Hepper & J.R.I. Wood and *Ozoroa sphaerocarpa* R. Fern. & A. Fern. as remedy for diarrhea [22]. Leaf and bark infusion of *S. cordatum* is taken orally for stomach ache in South Africa and Swaziland [1,5,7,23,24]. Bark infusion of *S. cordatum* is used against TB in South Africa and Zimbabwe [1,25,26,27]. Bark and root infusion of *S. cordatum* is applied topically on wounds in Kenya and South Africa [23,28,29]. In South Africa, bark, fruits, leaves and roots are taken orally for wounds in the mouth and ulcers [30,31]. In South Africa, bark decoction of *S. cordatum* is applied topically on burns or sores as monotherapy or mixed with bark of *Acacia burkei* Benth., *Ozoroa engleri* R. Fern. & A. Fern., *Sclerocarya birrea* (A. Rich.) Hochst., *Tabernaemontana elegans* Stapf. and *Lippia javanica* (Burm. f.) Spreng. [32]. Bark infusion of *S. cordatum* is used against STIs as monotherapy, mixed with *S. birrea* or mixed with *Aloe marlothii* A. Berger, *Hypoxis hemerocallidea* Fisch., C.A. Mey & Avé-Lall, *Senecio serratuloides* DC. and *S. birrea* [33,34]. Bark decoction of *S. cordatum* is used as emetics in South Africa and Swaziland [1,5,24,25,26]. In Tanzania and Uganda, the bark or leaf decoction of *S. cordatum* is applied topically for skin rash [29,35,36]. Bark and leaf decoction of *S. cordatum* is taken orally mixed with leaves of *S. birrea* as remedy for gonorrhea [37]. Bark and leaf decoction of *S. cordatum* is taken orally against colds in South Africa and Kenya [20,23]. In South Africa, bark, leaf or root infusion of *S. cordatum* is used against amenorrhea [23,28], chest complaints [25], colds [23], fever [23], headache [23] and respiratory ailments [1,7]. In Tanzania, bark or leaf decoction of *S. cordatum* is used against herpes simplex and zoster [35,36], while, in Tanzania and Zambia, bark or leaf decoction is used against malaria [38,39,40]. In Uganda, bark, leaf or root infusion of *S. cordatum* is used against anemia, hepatic jaundice [41] and dry cough [29]. 

## 4. Phytochemistry

*Syzygium cordatum* is characterized by different secondary metabolites such as anthocyanidin, carboxylic acid, catechin, essential oil components, hydroxycinnamic acid, leucoanthocyanidin, phenolic acids, phytosterols, simple sugars and triterpenoids (Table 3). Candy et al. [42] identified friedelin, epifriedelinol, β-sitosterol, tannin, arjunolic acid, ellagic acid (hexahydroxydiphenic acid), glucose and gallic acid using infrared spectroscopy (IR), co-chromatography (CC), two-dimensional chromatography (TDC) techniques and mass spectrometry (MS) from wood and bark of *S. cordatum*. Candy et al. [42] also isolated leucodelphinidin, leucocyanidin, deIphinidin and cyanidin from the bark and leaves of *S. cordatum*. Ndhlala et al. [43] evaluated the phenolic compound content and profiles of *S. cordatum* using the colorimetric methods and high-performance liquid chromatography (HPLC) and identified p-coumaric acid, vanillic acid, protocatechuic acid and caffeic acid from the fruits of the species. Chalannavar et al. [44] extracted essential oil from the leaves of *S. cordatum* by the hydrodistillation procedure and identified the components by gas chromatography (GC/FID) and mass spectrometry (GC/MS). The main constituent essential oil components (> 3.0%) were: methane, bis (2-chloroethoxy) (3.8%), isopentyloxyethyl acetate (5.0%), ethane, 2-chloro-1, -bis(2 chloroethoxy) (6.3%), n-hexadeconic acid (7.3%), 2,3-butanediol diacetate (13.3%) and 6,10,14-trimethylpentadecane-2-one (14.4%) [44]. Cordier et al. [45] identified caffeic acid, cinnamic acid, epigallocatechin, gallic acid, hesperidin and sinapic acid from bark extracts of *S. cordatum* using thin layer chromatography (TLC). Maliehe et al. [46,47] identified betulinic acid from fruit and seed extracts of *S. cordatum* using thin layer chromatography (TLC).

Alkaloids, anthracenoside aglycones (emodols), anthraquinones, cardiac glycosides, flavonoids, glucosides, phenols, saponins, reducing sugars, steroids, tannins, terpenoids and triterpenoids have been identified from bark, fruits, leaves and seeds extracts of *S. cordatum* [43,45,46,47,48,49,50,51,52,53]. Some of these phytochemicals have been quantified (Table 4) and these include flavonoids, flavonols, gallotannin, phenolics, proanthocyanidin and tannins [43,45,46,47,50].

## 5. Pharmacological Activities

Several pharmacological activities of *S. cordatum* have been reported in the literature justifying some of the medicinal uses of the species. These pharmacological activities include antibacterial [16,46,47,48,50,52,53,54], antifungal [50,53,55,56,57,58], antidiarrheal [22,47,51,52], anti-sexually transmitted infections [33,34], antidiabetic [51,59], anticholinesterase [50], anti-inflammatory [50,60], antileishmanial [61], antioxidant [45,48,60,62], antiplasmodial [39,63,64] and anti-proteus [65].

### 5.1. Antibacterial Activity

Samie et al. [54] evaluated the antibacterial activities of methanol, acetone and hexane bark and leaf extracts of *S. cordatum* against *Aeromonas hydrophila*, *Bacillus cereus*, *Bacillus pumilus*, *Bacillus subtilis*, *Enterobacter cloacae*, *Enterococcus fecalis*, *Escherichia coli*, *Klebsiella pneumoniae*, *Pantoea agglomerans*, *Proteus mirabilis*, *Pseudomonas aeruginosa*, *Salmonella enterica*, *Serratia marcescens*, *Shigella flexneri* and *Staphylococcus aureus* using the disc diffusion and the microdilution methods with gentamicin as positive control. The extracts exhibited activities with zones of inhibition ranging from 8.0 mm to 22.0 mm which was comparable to zone of inhibition of 18–30 mm exhibited by gentamicin (30 µg), the control. The MIC values ranged from 0.2 mg/mL to > 12.0 mg/mL. Mathabe et al. [16] evaluated antibacterial activities of acetone, ethanol, methanol and aqueous extracts of *S. cordatum* against *Escherichia coli*, *Salmonella typhyi*, *Shihella boydii*, *Shigella dysenterae*, *Shigella flexneri*, *Shigella sonnei*, *Staphylococcus aureus* and *Vibrio cholerae* using agar-well diffusion and serial dilution assays with dimethyl sulfoxide (DMSO) as negative control, and nalidixic acid, erythromycin and cotrimoxazole as positive controls. The extracts showed activities with zone of inhibition ranging from 11.7 mm to 25.0 mm against all the tested pathogens. The minimum inhibition concentration (MIC) values against the pathogens ranged from 0.08 mg/mL to 0.31 mg/mL [16]. Pallant and Steenkamp [48] evaluated antibacterial activities of methanol and water bark extracts of *S. cordatum* against *Haemophilis influenzae*, *Klebsiella pneumoniae*, *Mycobacterium smegmatis*, *Staphylococcus aureus* and *Streptococcus pneumoniae* using the disc diffusion and broth microdilution assays with ampicillin as the positive control. The aqueous extract exhibited activities against *Haemophilis influenzae* and *Staphylococcus aureus* with zone of inhibition ranging from 21.2 ± 0.2 mm to 22.5 ± 0.9 mm which was comparable to the zone of inhibition of 21.2 ± 0.4 mm to 39.7 ± 0.2 mm exhibited by ampicillin (30 µg), the control. The MIC values of both extracts against *Staphylococcus aureus* was 0.5 mg/mL [48]. 

Mulaudzi et al. [50] evaluated antibacterial activities of petroleum ether, dichloromethane, ethanol and water leaf extracts of *S. cordatum* against *Bacillus subtilis*, *Escherichia coli*, *Klebsiella pneumoniae* and *Staphylococcus aureus* using microdilution assay with neomycin as a positive control. The extracts exhibited activities with MIC values ranging from 0.01 µg/mL to 6.3 µg/mL [50]. Maliehe et al. [46] evaluated antibacterial activities of fruit and seed extracts of *S. cordatum* against bacteria causing gastro-intestinal tract infections which included *Bacillus cereus, Enterococcus hirae*, *Escherichia coli*, *Klebsiella pneumoniae*, *Pseudomonas aeruginosa*, *Salmonella typhimurium*, *Staphylococcus aureus*, *Vibrio fluvialis* and *Vibrio vulnificus* using agar dilution and serial microdilution methods with DMSO and ciprofloxacin as negative and positive controls, respectively. Pulp extract exhibited the lowest MIC values ranging from 3.1 mg/mL to 6.3 mg/mL and minimum bactericidal concentration (MBC) values of 3.1 mg/mL to 12.5 mg/mL against *Bacillus cereus*, *Enterococcus hirae*, *Klebsiella pneumoniae*, *Pseudomonas aeruginosa* and *Staphylococcus aureus*. The seed extract exhibited MIC values ranging from 3.1 mg/mL to 25.0 mg/mL and MBC values ranging from 12.5 mg/mL to 50.0 mg/mL against *Bacillus cereus*, *Enterococcus hirae*, *Klebsiella pneumoniae*, *Pseudomonas aeruginosa* and *Staphylococcus aureus* [46]. Maliehe et al. [47] evaluated antibacterial activities of *S. cordatum* pulp and seed extracts against *Bacillus cereus*, *Enterococcus hirae*, *Escherichia coli*, *Pseudomonas aeruginosa*, *Staphylococcus aureus* and *Vibrio vulnificus* using the microdillution method with DMSO and ciprofloxacin as negative and positive controls, respectively. Extracts exhibited activities with MIC and MBC values ranging from 3.1 mg/mL to 50.0 mg/mL which was comparable to MIC and MBC values of ciprofloxacin, the positive control ranging from 1.6 mg/mL to 12.5 mg/mL [47]. Maliehe et al. [52] evaluated antibacterial activities of methanol pulp extract of *S. cordatum* against *Bacillus cereus, Enterococcus hirae, Escherichia coli*, *Klebsiella pneumoniae, Pseudomonas aeruginosa, Salmonella typhimurium, Staphylococcus aureus, Vibrio fluvialis* and *Vibrio vulnificus* using serial microdilution method with DMSO and ciprofloxacin as negative and positive controls, respectively. The extract exhibited activities with MIC and MBC values ranging from 3.1 mg/mL to 6.3 mg/mL and 3.1 mg/mL to 12.5 mg/mL, respectively; and these values are comparable to MIC and MBC values of the control which ranged from 1.6 mg/mL to 3.1 mg/mL and 3.1 mg/mL to 12.5 mg/mL, respectively [52].

Nciki et al. [53] evaluated antibacterial activities of aqueous and dichloromethane:methanol (1:1) bark extracts of *S. cordatum* against *Brevibacterium agri*, *Brevibacterium linens*, *Escherichia coli*, *Propionibacterium acnes*, *Pseudomonas aeruginosa*, *Staphylococcus aureus* and *Staphylococcus epidermidis* using the micro-titer plate dilution assay with ciprofloxacin as a positive control. The antibacterial interaction of *S. cordatum* used in combination with S. birrea and also in combination with *A. burkei*, *O. engleri*, *S. birrea*, *T. elegans* and *L. javanica* was determined by calculating the sum of the fractional inhibitory concentrations (∑FIC) against *Pseudomonas aeruginosa*, *Staphylococcus aureus* and *Staphylococcus epidermidis*. The ∑FIC value was used to determine if the combined plants had synergistic effect (∑FIC ≤ 0.5), additive (∑FIC > 0.5−1.0), non-interactive (∑FIC > 1.0 ≤ 4.0) or antagonistic (∑FIC > 4.0) [53]. The extracts showed activities with MIC values ranging from 60.0 µg/mL to > 8000.0 µg/mL which was much higher than MIC values of 0.1 µg/mL to 1.25 µg/mL exhibited by ciprofloxacin, the control. The combination of *S. cordatum* with *S. birrea* resulted in ∑FIC values ranging from 0.1 to 1.5, indicating synergistic to non-interactive effect, and with *A. burkei*, *O. engleri*, *S. birrea*, *T. elegans* and *L. javanica* resulted in ∑FIC values ranging from of 0.57 to 2.45, indicating additive to non-interactive effects [53]. Antibacterial evaluations of *S. cordatum* combined with other species showed some evidence of synergistic and additive effects [53], thus supporting the traditional method of preparing these combined remedies for burns [32], diarrhea [22], gonorrhea [37], STIs [34] and sores [32].

Sibandze et al. [22] evaluated antibacterial activities of mono-extracts of *S. cordatum* bark or in combination with bark extracts of *B. salicina* and *O. sphaerocarpa* against a diarrhea-causing pathogen, *Escherichia coli* with ciprofloxacin as a positive control. Mono-extracts of *S. cordatum* exhibited activities with MIC value of 1.4 mg/mL, the combination between *S. cordatum* and *O. sphaerocarpa* gave MIC value of 0.3 mg/mL and that between *S. cordatum* and *B. salicina* gave MIC value of 1.0 mg/mL. The triple combination exhibited MIC value of 0.4 mg/mL. These findings support the rationale by traditional healers to use the bark of *S. cordatum, B. salicina* and *O. sphaerocarpa* in combination for the treatment of diarrhea in Swaziland [22].

Van Vuuren and Naidoo [33] evaluated anti-sexually transmitted infections activities of dichloromethane and methanol (1:1) and aqueous leaf extracts of *S. cordatum* against *Candida albicans, Gardnerella vaginalis, Neisseria gonorrhoeae, Oligella ureolytica, Trichomonas vaginalis* and *Ureaplasma urealyticum* with ciprofloxacin and amphotericin B as positive controls. The extracts exhibited activities with MIC values ranging from 0.1 mg/mL to > 16.0 mg/mL while the controls, ciprofloxacin and amphotericin B exhibited MIC values of 0.04 µg/mL to 0.6 µg/mL and 2.5 µg/mL, respectively [33]. Similarly, Naidoo et al. [34] evaluated anti-sexually transmitted infections activities of aqueous and dichloromethane and methanol (1:1) bark extracts of *S. cordatum* against *Candida albicans, Gardnerella vaginalis*, *Neisseria gonorrhoeae*, *Oligella ureolytica*, *Trichomonas vaginalis* and *Ureaplasma urealyticum* using the micro-titer plate dilution method with ciprofloxacin and amphotericin B as positive controls. The anti-sexually transmitted infections interaction of *S. cordatum* used in combination with *S. birrea* and also in combination with *H. hemerocallidea, S. birrea, S. serratuloides* and *A. marlothii* was determined by calculating the sum of the fractional inhibitory concentrations (∑FIC) against *Candida albicans, Gardnerella vaginalis, Neisseria gonorrhoeae*, *Oligella ureolytica*, *Trichomonas vaginalis* and *Ureaplasma urealyticum*. The extracts exhibited activities with MIC values ranging from 0.3 mg/mL to 8.0 mg/mL while the controls, ciprofloxacin (0.01 mg/mL) and amphotericin B (0.1 mg/mL) exhibited MIC values of 0.04 µg/mL to 0.6 µg/mL and 2.5 µg/mL, respectively. The combination of *S. cordatum* with *S. birrea* resulted in MIC values ranging from 0.3 mg/mL to > 16.0 mg/mL while ∑FIC values ranged from 0.42 to 5.0. The combination of *S. cordatum* with *H. hemerocallidea, S. birrea, S. serratuloides* and *A. marlothii* resulted in MIC values ranging from 0.8 mg/mL to > 16.0 mg/mL while ∑FIC values ranged from 0.7 to 24.5 [34]. These results corroborate the potential of *S. cordatum* in the treatment and management of STIs and, therefore, support its traditional uses against this disease in South Africa [33,34]. Anti-sexually transmitted infections interaction evaluations of *S. cordatum* combined with other species showed some evidence of synergy [34], thus supporting the traditional method of preparing these combined remedies for STIs in South Africa [34].

### 5.2. Antifungal Activity

Steenkamp et al. [55] evaluated antifungal activities of methanol and water bark extracts of *S. cordatum* against *Candida albicans* using plate-hole diffusion assay with amphotericin B as the positive control. The extract exhibited activity with MIC values ranging from 0.8 mg/mL to 3.8 mg/mL. Pallant and Steenkamp [48] evaluated antifungal activities of methanol and water bark extracts of *S. cordatum* against *Candida albicans* using the disc diffusion and broth microdilution assays with amphotericin B as the positive control. Both methanol and water extracts exhibited activities with zone of inhibition ranging from 21.7 ± 0.7 mm to 24.3 ± 0.2 mm which was comparable to zone of inhibition of 33.5 ± 3.2 mm exhibited by amphotericin B (20 µg), the control. The MIC values of both extracts were > 1 mg/mL [48]. Mangoyi and Mukanganyama [56] evaluated the antifungal activities of bark and leaf extracts of *S. cordatum* against *Candida albicans* and *Candida krusei* using the agar disc diffusion and broth dilution methods with miconazole as the positive control. The extracts exhibited activities with zone of inhibition ranging from 12.0 ± 0.1 mm to 15.0 ± 0.1 mm, and MIC and minimum fungicidal concentration (MFC) values ranging from 0.6 mg/mL to 2.5 mg/mL against both species. The zone of inhibition exhibited by miconazole, the control, was 20.0 ± 0.8 mm to 22.6 ± 0.7 mm, and the MIC and MFC values ranged from 0.3 mg/mL to 0.6 mg/mL [56]. 

Mulaudzi et al. [50] evaluated antifungal activities of petroleum ether, dichloromethane, ethanol and water leaf extracts of *S. cordatum* against *Candida albicans* using microdilution assay with amphotericin B as the control. The extracts exhibited activities with MIC and MFC values ranging from 0.2 µg/mL to 6.3 µg/mL and 0.4 µg/mL to 12.5 µg/mL, respectively [50]. Masangwa et al. [57] evaluated antifungal activities of acetone, ethyl acetate and water leaf extracts of *S. cordatum* against *Colletotrichum lindemuthianum* and *Colletotrichum dematium* using the agar disc infusion and micro-titer double-dilution techniques with DMSO and the fungicide fludioxonil + mefenoxam (as commercial product Celest^®^ XL) as negative and positive controls, respectively. The same extracts were then tested for antifungal activity in vivo as seed treatments against anthracnose disease. All extracts showed activities against the tested fungi with MIC values ranging from 0.8 mg/mL to 6.3 mg/mL and the MIC value of the positive control, Celest^®^ XL was 0.1 mg/mL. The extracts reduced anthracnose disease of bean and cowpea and therefore, are potential seed treatments in anthracnose disease control [57]. Nciki et al. [53] evaluated antifungal activities of aqueous and dichloromethane and methanol (1:1) bark extracts of *S. cordatum* against *Candida albicans*, *Microsporum canis* and *Trichophyton mentagrophytes* using the micro-titer plate dilution assay with amphotericin B as a positive control. The extracts showed weak activities with MIC values ranging from 380.0 µg/mL to > 8000.0 µg/mL which was much higher than MIC values of 0.01 µg/mL to 0.1 µg/mL exhibited by amphotericin B (100 µg/mL), the control [53]. Njoki et al. [58] evaluated antifungal activities of aqueous bark extract of *S. cordatum* against *Aspergillus flavus* using disc diffusion and broth dilution methods. The extract exhibited activities with the zone of inhibition ranging from 9.5 ± 0.7 mm to 17.0 ± 1.3 mm which was comparable to the zone of inhibition ranging from 17.2 ± 0.4 mm to 22.0 ± 0.6 mm exhibited by the positive control at 250 mg/mL [58].

### 5.3. Antidiarrheal Activity

Deliwe and Amabeoku [51] evaluated antidiarrheal activities of leaf aqueous extract of *S. cordatum* in male albino mice using castor oil-induced diarrheal test. The extract significantly reduced the number of diarrheal episodes, decreased the stool mass and delayed the onset of castor oil-induced diarrhea in mice [51]. Maliehe et al. [47] evaluated antidiarrheal activities of *S. cordatum* pulp and seed extracts using the castor oil-induced rat model. The *S. cordatum* fruit-pulp and seed extracts both reduced the number of wet stools, total stools and onset time generally in comparison to the negative control (distilled water). The *S. cordatum* fruit-pulp and seed extracts, in a dose-related manner (400 mg/kg of rat), exerted the antidiarrheal properties by reducing intestinal motility [47]. Maliehe et al. [52] evaluated the antidiarrheal and antimotility activities of methanolic pulp extracts of *S. cordatum* using castor oil-induced diarrhea model in rats. The fruit pulp extract reduced the number of wet stools, total number of stools and onset time generally in comparison to the negative control (distilled water). Fruit pulp extract, in a dose-related manner (400 mg/kg of rat), exerted the antidiarrheal property by reducing intestinal motility as well [52]. These findings lend credence to the traditional uses of *S. cordatum* as remedy for diarrhea [1,5,15,16,17,18,19,20,22], dysentery [15] and gastro-intestinal complications [21].

### 5.4. Antidiabetic Activity

Musabayane et al. [59] evaluated the hypoglycaemic effect of *S. cordatum* leaf extract in non-diabetic and streptozotocin-induced diabetic rats. Oral glucose tolerance tests were conducted in non-diabetic and streptozotocin-diabetic rats using orally administered glucose at 1.4 g/100 g body weight followed by either the leaf extract at 6 mg/100 g body weight or subcutaneous injection of metformin at 50 mg/100 g. Weekly plasma glucose and terminal hepatic glycogen concentrations were recorded in control streptozotocin-diabetic rats and diabetic rats orally treated with the leaf extract once every third day for four weeks. Administration of the leaf extract decreased plasma glucose from 7.7 ± 0.9 mmol/L to 3.7 ± 0.6 mmol/L and 21.1 ± 2.2 mmol/L to 12.5 ± 1.8 mmol/L in 2.5 h in non-diabetic and streptozotocin-diabetic rats, respectively [59]. Deliwe and Amabeoku [51] evaluated antidiabetic activities of leaf aqueous extract of *S. cordatum* using streptozotoxin-induced diabetes in Wistar rats. Both the extract at 12.5 mg/kg to 50.0 mg/kg and chlorpropamide at 250.0 mg/kg significantly lowered the blood glucose levels in both normal and streptozotoxin-induced diabetic rats. Since chlorpropamide is used to treat diabetes by stimulating insulin secretion from pancreatic beta cells and promoting peripheral glucose uptake and utilization, it is probable that *S. cordatum* acts in a similar manner [51]. Therefore, *S. cordatum* leaf extracts could be effective in mild diabetes mellitus or in cases of glucose tolerance impairment but might be less effective in severe hyperglycaemia.

### 5.5. Anticholinesterase Activity

Mulaudzi et al. [50] evaluated acetylcholinesterase (AChE) enzyme inhibitory effects of petroleum ether, dichloromethane, ethanol and water extracts of *S. cordatum*. The methanolic and water extracts showed high AChE inhibitory activities of 88.7% and 85.3%, respectively, with median inhibitory concentration (IC_50_) values of 0.2 ± 0.02 mg/mL and 0.3 ± 0.01 mg/mL, respectively [50]. 

### 5.6. Anti-Inflammatory Activity

Mulaudzi et al. [50] evaluated anti-inflammatory activities of petroleum ether, dichloromethane, ethanol and water extracts of *S. cordatum* by evaluating their ability to inhibit cyclooxygenase-1 and 2 (COX-1 and COX-2) enzymes. Petroleum ether and dichloromethane extracts exhibited high inhibition activity towards both COX-1 and COX-2 exceeding 75% [50]. Mzindle [60] evaluated anti-inflammatory activities of methanol and water extracts of *S. cordatum* using the lipoxygenase inhibitor screening assay with nordihydroguaiaretic acid as a positive control. The methanol and water extracts inhibited lipoxygenase enzyme by 78.6 ± 3.6% and 40.5 ± 6.7%, respectively, which was lower than 122% and 129% inhibition demonstrated by nordihydroguaiaretic acid, the control [60]. Mzindle [60] also evaluated the wound healing activities of ethanol and water extracts of *S. cordatum* using the scratch wound assay. The migration rate of the extracts ranged from 23.3 ± 18.1% to 60.2 ± 0.0% when compared to the untreated cells with a percentage migration rate of 24%. These findings support the traditional use of *S. cordatum* in managing inflammatory ailments and diseases such as burns, sores, ulcers and wounds [1,23,28,29,30,31] and other problems that result in cell injury and death.

### 5.7. Antileishmanial Activity

Bapela et al. [61] evaluated antileishmanial activities of dichloromethane and methanol leaf extracts of *S. cordatum* against *Leishmania donovani*. The dichloromethane extracts displayed high inhibitory effects on the growth of amastigote forms of *Leishmania donovani* with IC_50_ values of 5.0 μg/mL. Bapela et al. [66] demonstrated that most of the non-polar extracts of medicinal plants used in the treatment of malaria also possess significant antiplasmodial activities, and, therefore, likely have antileishmanial properties as both malaria and leishmaniasis are protozoal infections sharing several unique metabolic pathways. Therefore, findings of this research imply that *S. cordatum* extracts may have potential as antileishmanial agents.

### 5.8. Antioxidant Activity

Pallant and Steenkamp [48] evaluated the antioxidant activities of methanol and water bark extracts of *S. cordatum* using the Trolox equivalent antioxidant capacity (TEAC) and free radical ABTS (2,2′-azinobis(3-ethylbenzothiazoline-6-sulfonic acid) assays. The antioxidant assay showed strong ABTS free radical scavenging activity by both extracts with TEAC values of 1.95 and 0.80, respectively. In comparison, the positive control for the assay, ascorbic acid, had a TEAC value of 2.45 [48]. Cordier et al. [45] evaluated the antioxidant activities of aqueous and methanolic bark extracts of *S. cordatum* in an in vitro oxidative stress model using the antioxidant capacity and by assessing the free radical scavenging activity using DPPH (2,2-diphenyly-1-picrylhydrazyl) assay. The antioxidant activity TEAC values ranged from 0.8 ± 0.0 to 2.8 ± 0.0 Trolox equivalents while DPPH values ranged from 0.7 ± 0.0 to 3.0 ± 0.1 Trolox equivalents. These values were comparable to TEAC values of 2.8 ± 0.0 Trolox equivalents and DPPH values of 1.7 ± 0.0 Trolox equivalents exhibited by the standard, ascorbic acid. Free radical-induced generation of reactive oxygen species (up to 80%), lipid peroxidation (up to 200%) and apoptosis (up to 60%) was successfully reduced by the extracts of *S. cordatum* [45]. Kucich and Wicht [62] evaluated the total antioxidant capacity (H-ORAC_FL+_L-ORAC_FL_). These authors obtained the following results: H-ORAC_FL+_ (77.0 ± 1.5 μmol Trolox equivalent/g fresh weight) and L-ORAC_FL_ (48.3 ± 2.4 μmol Trolox equivalent/g fresh weight) and total antioxidant capacity (TAC) value of 125.4 μmol Trolox equivalent/g fresh weight [62]. Mzindle [60] evaluated antioxidant activities of methanol and water extracts of *S. cordatum* using the DPPH assay with rutin as a positive control. The extracts showed free radical scavenging abilities ranging from 48.1 ± 1.5% to 99.0 ± 0.2%, while rutin exhibited free radical scavenging abilities ranging from 27.4 ± 1.4% to 95.3 ± 0.5% [60]. The documented antioxidant activities [45,48,60,62] are probably due to flavonoids and phenolics that have been isolated from the species [43,45,46,47,50].

### 5.9. Antiplasmodial Activity

Clarkson et al. [63] evaluated antiplasmodial activities of *S. cordatum* aqueous, dichloromethane, dichloromethane and methanol (1:1) leaf and twig extracts against *Plasmodium falciparum* using the parasite lactate dehydrogenase (pLDH) assay. *Syzygium cordatum* dichloromethane and methanol (1:1) extracts showed weak activities with IC_50_ values ranging from 14.7 µg/mL to 48.3 µg/mL. Bapela et al. [64] evaluated antiplasmodial activities of dichloromethane leaf extracts of *S. cordatum* using the [3H]hypoxanthine incorporation assay using chloroquine sensitive (NF54) strain of *Plasmodium falciparum* as the test organism. The extract showed activity with IC_50_ value of 6.2 μg/mL [64]. Nondo et al. [39] evaluated antiplasmodial activities of ethanol stem bark extract of *S. cordatum* against chloroquine-resistant *Plasmodium falciparum* (Dd2) using the parasite lactate dehydrogenase method. The extract inhibited the growth of the chloroquine-resistant Dd2 malaria parasite strains by 55.5 ± 13.4% [39]. These findings support the use of *S. cordatum* for the treatment of fever in South Africa [23] and malaria in Tanzania [38,39].

### 5.10. Anti-Proteus Activity

Cock and van Vuuren [65] evaluated the activities of methanol and water bark and leaf extracts of *S. cordatum* against *Proteus mirabilis* and *Proteus vulgaris* using modified disc diffusion method with ampicillin and chloramphenicol as positive controls and distilled water and methanol as negative controls. The extracts exhibited activities against tested pathogens with zone of inhibition ranging from 10.0 ± 1.0 mm to 13.7 ± 0.6 mm and the MIC value ranged from 49.0 µg/mL to 1325.0 µg/mL [65].

### 5.11. Cytotoxicity Activity

Verschaeve et al. [66] evaluated mutagenic and antimutagenic activities of dichloromethane extracts of leaf extracts of *S. cordatum* in *Salmonella/*microsome and micronucleus tests. None of the extracts tested in the Ames test were found to induce mutations or to modify the effect of the mutagen 4-nitroquinoline-oxide (4NQO). In the micronucleus test, the extracts significantly lowered the effect of the mutagen mitomycin C (MMC) [66]. Sibandze et al. [22] evaluated the cytotoxicity of combined effect of bark extracts of *S. cordatum, B. salicina* and *O. sphaerocarpa* against human kidney epithelial cells, using the MTT (3-[4,5-dimethylthiazol-2yl]-2,5diphenyltetrazolium bromide) cellular viability assay. The triple combination had a favorable cytotoxicity profile with an IC_50_ value of 155.8 ± 11.9 µg/mL [22]. Mulaudzi et al. [50] evaluated the cytotoxicity activities of petroleum ether, dichloromethane, ethanol and water extracts of *S. cordatum* by evaluating the mutagenicity using the *Salmonella*/microsome assay using the plate-incorporation procedure with *Salmonella typhimurium* tester strains TA98, TA100 and TA102 with and without enzyme (S9) bioactivation. None of the extracts showed mutagenic effects [50]. Cordier et al. [45] evaluated the cytotoxicity activities of aqueous and methanolic bark extracts of *S. cordatum* in C2C12 myoblasts, 3T3-L1 pre-adipocytes, normal human dermal fibroblasts and U937 macrophage-like cells using the neutral red uptake assay. The extracts were most toxic to the 3T3-L1 with IC_50_ values ranging from 25.0 ± 1.0 µg/mL to 74.6 ± 1.0 µg/mL and C2C12 with IC_50_ values ranging from 20.5 ± 1.1 µg/mL to 95.6 ± 1.1 µg/mL and but not cytotoxic in the U937 and normal human dermal fibroblasts cultures with IC_50_ values > 100 μg/mL [45]. Naidoo et al. [34] evaluated cytotoxicity of the dichloromethane and methanol (1:1) and aqueous leaf extracts of *S. cordatum* using the MTT cellular viability assay. The aqueous and organic extracts were non-toxic, they exhibited cellular viability at 104.0 ± 0.8 µg/mL and 102.0 ± 0.8 µg/mL, respectively against the human kidney epithelial cell line [34]. Bapela et al. [64] evaluated cytotoxicity activities of leaf extracts of *S. cordatum* against mammalian L-6 rat skeletal myoblast cells with podophyllotoxin as a control. The extract demonstrated IC_50_ value of 65.7 μg/mL and selectivity index value of 10.7 which was considered to be toxic to rat skeletal myoblast L6 cells [64].

Nondo et al. [67] evaluated the cytotoxic activities of ethanol stem bark extract of *S. cordatum* using MTT assay on LLC-MK2 monkey kidney epithelial cells. The extract was non-cytotoxic and exhibited 50% cytotoxic concentration (CC_50_) values above 200 μg/mL [67]. Bapela et al. [61] evaluated cytotoxicity activities of dichloromethane and methanol leaf extracts of *S. cordatum* by assessing the inhibition of mammalian cell growth by cultivating rat skeletal myoblast L6 cells in the presence of different extracts covering a concentration range from 0.002 to 100.0 μg/mL in 96 well culture plates with podophyllotoxin as a positive control. The methanol and dichloromethane extracts exhibited IC_50_ values of 53.8 μg/mL and 65.7 μg/mL, respectively, which were much higher than IC_50_ value of 0.007 μg/mL exhibited by podophyllotoxin, the control [61]. Maliehe et al. [52] evaluated the cytotoxicity activities of methanolic pulp extracts of *S. cordatum* using the MTT assay and exhibited IC_50_ value of 92.0 μg/mL. Mzindle [60] evaluated cytotoxicity of methanol and water leaf extracts of *S. cordatum* using MTT assay using 3T3 NIH fibroblast cells by treating them with various concentrations of the extracts. The extracts exhibited 100% to 120% viability, indicating that the extracts were not toxic to the cells [60].

### 5.12. Toxicity

Cock and van Vuuren [65] evaluated toxicity of methanol and water bark and leaf extracts of *S. cordatum* using a modified *Artemia franciscana* nauplii lethality assay with sea water as the negative control. The extracts are non-toxic as the LC_50_ values were above that of the negative control. Deliwe and Amabeoku [51] evaluated acute toxicity of leaf aqueous extract of *S. cordatum* using male albino mice. The extract was administered orally to mice in graded doses of 200, 400, 800, 1200, 1600, 2000, 2400, 2800, 3200, 3600 and 4000 mg/kg. The control group received 0.3 mL physiological saline orally; both the test and control animals were allowed access to food and water; and the animals were observed for five days for any deaths or acute toxicity symptoms such as hypoactivity, piloerection and salivation. The median lethal dose (LD_50_) value obtained for the extract was over 4000 mg/kg orally. The relatively high LD_50_ value obtained for the extract shows that *S. cordatum* is non-toxic to mice [51]. Nondo et al. [67] evaluated the toxicity activities of ethanol stem bark extract of *S. cordatum* using the brine shrimp (*Artemia salina* L.) lethality assay. The brine shrimp lethality assay demonstrated LC_50_ value of 99.9 μg/mL and, therefore, non-toxic [67]. Further toxicological evaluations of *S. cordatum* should be carried out as powdered bark of the species is sometimes used as a fish poison [12,25]. Bark extracts of *S. cordatum* poisons small fish and turns water blue for a week although the poison is not potent for more than three days [25]. Therefore, it is important to determine if any toxicological effects can occur from its chronic or subchronic usage given the widespread use of *S. cordatum* as herbal medicine.

## 6. Conclusions

Pharmacological studies of the various parts of *S. cordatum* have supported and justified the traditional uses and ethnopharmacological importance of the species. The antimicrobial, anti-inflammatory, antioxidant and antiplasmodial activities are consistent with the use of *S. cordatum* in the treatment of burns, chest complaints, colds, cough, fever, gastro-intestinal problems, herpes simplex or zoster, malaria, respiratory complaints, STIs, skin rash, sores, TB and wounds. The anthocyanidin, essential oils, flavonoids, leucoanthocyanidin, phenolics, phytosterols and triterpenoids appear to be the major plant derivatives and active ingredients in the bark, fruits, leaves and seed extracts of *S. cordatum*. There are few to no pharmacological evaluations done to date focusing on the biological effects of the phytochemical compounds isolated from *S. cordatum*. Therefore, future research should focus on pharmacokinetics and clinical research of *S. cordatum* products and compounds. This research should be complemented by experimental animal studies, randomized clinical trials and target-organ toxicity studies involving *S. cordatum* products, compounds and its derivatives. Therefore, future research should identify the bioactive components, details of their molecular modes or mechanisms of action, pharmacokinetics and physiological pathways for specific bioactive compounds and plant parts of *S. cordatum*.

## Figures and Tables

**Table 1:** Medicinal applications of *Syzygium cordatum* in east and southern Africa.

Use	Plant Parts Used	Country Practiced	References
Amenorrhea	Bark and roots	South Africa	[23,28]
Anemia	Bark and leaves	Uganda	[40]
Burns	Bark infusion taken orally mixed with *Sclerocarya birrea* (A. Rich.) Hochst.	South Africa	[1]
Chest complaints	Bark	South Africa	[25]
Colds	Bark and leaves	Kenya, South Africa	[20,23]
Cough	Roots	Uganda	[29]
Diarrhea	Bark, leaves and roots	Kenya, Malawi, Namibia, South Africa, Zambia	[1,5,15,16,17,18,19,20]
Diarrhea	Stem bark infusion taken orally mixed with *Breonadia salicina* (Vahl) Hepper & J.R.I. Wood and *Ozoroa sphaerocarpa* R. Fern. & A. Fern.	Swaziland	[22]
Dysentery	Roots	Malawi	[15]
Emetics	Bark	South Africa, Swaziland	[1,5,24,25,26]
Fever	Leaves	South Africa	[23]
Gastro-intestinal complications	Leaves	Kenya	[21]
Gonorrhea	Bark and leaf infusion taken orally mixed with *S. birrea*	South Africa	[37]
Headache	Bark and roots	South Africa	[23]
Herpes simplex	Bark and leaves	Tanzania	[35,36]
Herpes zoster	Bark and leaves	Tanzania	[35,36]
Malaria	Leaves, roots and stem bark	Tanzania, Zambia	[38,39,40]
Pre-hepatic jaundice	Bark and leaves	Uganda	[41]
Respiratory ailments	Bark	South Africa	[1,7]
Sexually transmitted infections (STIs)	Bark	South Africa	[33,34]
STIs	Bark infusion taken orally mixed with *S. birrea*	South Africa	[34]
STIs	Bark infusion taken by mouth mixed with *Aloe marlothii* A. Berger, *Hypoxis hemerocallidea* Fisch., C.A. Mey & Avé-Lall, *Senecio serratuloides* DC. and *S. birrea*	South Africa	[34]
Skin rash	Bark and leaves	Tanzania, Uganda	[29,35,36]
Sores	Bark of S. cordatum applied topically as monotherapy or mixed with *Acacia burkei* Benth., *Ozoroa engleri* R. Fern. & A. Fern., *S. birrea*, *Tabernaemontana elegans* Stapf. and *Lippia javanica* (Burm. f.) Spreng.	South Africa	[32]
Stomach problems	Bark and leaf	South Africa, Swaziland	[1,5,7,23,24]
Tuberculosis	Bark	South Africa, Zimbabwe	[1,25,26,27]
Ulcer	Leaf and roots	South Africa	[31]
Wounds	Bark and roots	South Africa, Uganda	[23,28,29]
Wound in the mouth	Leaf, fruit and stem bark	South Africa	[30]

**Table 2:** Major disease or ailment categories reported.

Disease or Ailment Category	Number of Literature Reports
Gastro-intestinal disorders	14
Burns, sores and wounds	7
Colds, cough and respiratory ailments	5
Tuberculosis	4
Sexually transmitted infections (STIs)	3
Fever and malaria	3

**Table 3:** Chemical compounds isolated and characterized from *Syzygium cordatum.*

Compound	Plant Part	Isolation and Identification Method	Reference
**Anthocyanidin**			
Cyanidin	Bark, wood	CC; IR	[42]
DeIphinidin	Bark, wood	CC; IR	[42]
**Carboxylic acid**			
Cinnamic acid	Bark	TLC	[45]
**Catechin**			
Epigallocatechin	Bark	TLC	[45]
Flavanon glycoside			
Hesperidin	Bark	TLC	[45]
**Hydroxycinnamic acid**			
Sinapic acid	Bark	TLC	[45]
**Leucoanthocyanidin**			
Leucodelphinidin	Bark, leaves	IR	[42]
Leucocyanidin	Bark, leaves	IR	[42]
**Phenolic acids**			
Caffeic acid	Bark, fruits	HPLC; TLC	[43,45]
p-coumaric acid	Bark, fruits	HPLC; TLC	[43,45]
Ellagic acid	Bark, wood	CC; IR	[42]
Gallic acid	Bark, wood	CC; IR; TLC	[42,45]
Gallic acid-ellagic acid complex	Bark, wood	IR; TDC	[42]
Hexahydroxydiphenic acid	Bark, wood	IR; TDC	[42]
Protocatechuic acid	Fruits	HPLC	[43]
Vanillic acid	Fruits	HPLC	[43]
**Polyphenol**			
Tannin	Bark, wood	IR	[42]
**Phytosterol**			
β-sitosterol	Bark, wood	CC; IR	[42]
**Simple sugar**			
Glucose	Bark, wood	CC; IR	[42]
**Triterpenoids**			
Arjunolic acid	Bark, wood	IR; MS	[42]
Betulinic acid	Fruit	TLC	[46,47]
Epifriedelinol	Bark, wood	CC; IR	[42]
Friedelin	Bark, wood	CC; IR	[42]
**Essential oil components**			
Azulene (0.1%)	Leaves	GC/FID; GC/MS	[44]
2(4*H*)-benzofuranone (0.1%)	Leaves	GC/FID; GC/MS	[44]
2-butanone, 4-(acetyloxy)–(0.1%)	Leaves	GC/FID; GC/MS	[44]
Cedrol (0.1%)	Leaves	GC/FID; GC/MS	[44]
Diepi-.α.-cedrene epoxide (0.1%)	Leaves	GC/FID; GC/MS	[44]
1,2-epoxy-3-propyl acetate (0.1%)	Leaves	GC/FID; GC/MS	[44]
Ethane, 1,2-bis(2-chloroethoxy)–(0.1%)	Leaves	GC/FID; GC/MS	[44]
Glycine, *N*-acetyl–(0.1%)	Leaves	GC/FID; GC/MS	[44]
2-heptanone (0.1%)	Leaves	GC/FID; GC/MS	[44]
Hydrazine, 2-propenyl (0.1%)	Leaves	GC/FID; GC/MS	[44]
Isophytol (0.1%)	Leaves	GC/FID; GC/MS	[44]
Ledol (0.1%)	Leaves	GC/FID; GC/MS	[44]
Nonanoic acid (0.1%)	Leaves	GC/FID; GC/MS	[44]
3-penten-2-one, 4-phenyl–(0.1%)	Leaves	GC/FID; GC/MS	[44]
Propane, 1,1,2-trichloro–(0.1%)	Leaves	GC/FID; GC/MS	[44]
(Trimethylsilyl)diazomethane (0.1%)	Leaves	GC/FID; GC/MS	[44]
1,3-dioxan-4-one (0.2%)	Leaves	GC/FID; GC/MS	[44]
1,1-ethanediol, diacetate (0.2%)	Leaves	GC/FID; GC/MS	[44]
3-hexanol (0.2%)	Leaves	GC/FID; GC/MS	[44]
6-Isopropenyl-4,8a-dimethyl-1,2,3,5,6,7,8,8a-octahydro-naphthalen-2-ol (0.2%)	Leaves	GC/FID; GC/MS	[44]
Octadecanoic acid, methyl ester (0.2%)	Leaves	GC/FID; GC/MS	[44]
Silane (0.2%)	Leaves	GC/FID; GC/MS	[44]
*Trans*-Z-.α.-bisabolene epoxide (0.2%)	Leaves	GC/FID; GC/MS	[44]
1-eicosene (0.3%)	Leaves	GC/FID; GC/MS	[44]
Ethane, 1,1-dichloro (0.3%)	Leaves	GC/FID; GC/MS	[44]
Ethanesulfonyl chloride, 2-chloro (0.3%)	Leaves	GC/FID; GC/MS	[44]
Eudesma-4(14),11-diene (0.3%)	Leaves	GC/FID; GC/MS	[44]
2,5-hexanedione (0.3%)	Leaves	GC/FID; GC/MS	[44]
4-methylthiazole (0.3%)	Leaves	GC/FID; GC/MS	[44]
1,3,4-oxadiazole (0.3%)	Leaves	GC/FID; GC/MS	[44]
Oxirane, 2,3-dimethyl–(0.3%)	Leaves	GC/FID; GC/MS	[44]
5-undecanone (0.3%)	Leaves	GC/FID; GC/MS	[44]
3-hepten-2-one, 5-methyl (0.4%)	Leaves	GC/FID; GC/MS	[44]
Isoaromadendrene epoxide (0.5%)	Leaves	GC/FID; GC/MS	[44]
Phytol (0.5%)	Leaves	GC/FID; GC/MS	[44]
3-heptanol (0.7%)	Leaves	GC/FID; GC/MS	[44]
Naphthalene, 1,2,3,4,4a,5,6,8a-octahydro(1.α.,4a.β.,8a.α.)–(0.7%)	Leaves	GC/FID; GC/MS	[44]
9,12,15-octadecatrienoic acid, methyl ester, (Z,Z,Z)–(0.7%)	Leaves	GC/FID; GC/MS	[44]
Oxazole, trimethyl (0.8%)	Leaves	GC/FID; GC/MS	[44]
2,4-pentanedione (0.8%)	Leaves	GC/FID; GC/MS	[44]
Toluene (0.8%)	Leaves	GC/FID; GC/MS	[44]
3-decanone (0.9%)	Leaves	GC/FID; GC/MS	[44]
2,4-dimethyl-3-pentanol acetate (1.1%)	Leaves	GC/FID; GC/MS	[44]
3-heptanol, 3,6-dimethyl–(1.1%)	Leaves	GC/FID; GC/MS	[44]
Naphthalene, 1,6-dimethyl-4-(1-methylethyl)–(1.1%)	Leaves	GC/FID; GC/MS	[44]
Triacetin (1.1%)	Leaves	GC/FID; GC/MS	[44]
2-furanone (1.3%)	Leaves	GC/FID; GC/MS	[44]
Ethylene maleic anhydride (1.4%)	Leaves	GC/FID; GC/MS	[44]
*N*,*N*,*N*’,*N*’-tetraacetylethylenediamine (2.0%)	Leaves	GC/FID; GC/MS	[44]
Naphthalene, 1,2,3,4-tetrahydro-1,6-dimethyl-4-(1-methylethyl)-, (1S-cis) (2.1%)	Leaves	GC/FID; GC/MS	[44]
Hexadecanoic acid, methyl ester (2.7%)	Leaves	GC/FID; GC/MS	[44]
Ethene, chloro–(2.9%)	Leaves	GC/FID; GC/MS	[44]
Methane, bis (2-chloroethoxy) (3.9%)	Leaves	GC/FID; GC/MS	[44]
Isopentyloxyethyl acetate (5.0%)	Leaves	GC/FID; GC/MS	[44]
Ethane, 2-chloro-1,1-bis(2-chloroethoxy) (6.3%)	Leaves	GC/FID; GC/MS	[44]
*n*-hexadeconic acid (7.3%)	Leaves	GC/FID; GC/MS	[44]
2,3-butanediol diacetate (13.1%)	Leaves	GC/FID; GC/MS	[44]
6,10,14-trimethylpentadecane-2-one (14.4%)	Leaves	GC/FID; GC/MS	[44]

**Table 4:** Moisture content and phytochemical compound profiles isolated from *Syzygium cordatum* fruits and other plant parts.

Phytochemicals of Fruit (Peel, Pulp) and Other Parts	Values	Reference
Moisture content (pulp)	0.9%	[43]
Condensed tannin (leaf)	34.6 ± 6.0% LCE ^a^	[50]
Flavonols (peel)	8.1 ± 1.6 µg/g	[43]
Flavonols (pulp)	10.6 ± 0.2 µg/g	[43]
Proanthocyanidin dry matter (peel)	0.21 ± 0.05%	[43]
Proanthocyanidin dry matter (pulp)	0.26 ± 0.04%	[43]
Total flavonoid content (bark)	130.6 ± 9.5 to 334.0 ± 9.7 mg RU/g ^b^	[45]
Total flavonoids (leaf)	4.56 ± 0.1 µg CTE/g ^c^	[50]
Total gallotannin ((leaf)	34.6 ± 6.0 µg GAE/g ^d^	[50]
Total phenolics (peel)	13.04 ± 0.44 µg/g	[43]
Total phenolics (pulp)	20.6 ± 1.18 µg/g	[43]
Total phenolics (seed)	21.4 ± 1.4 µg/mL	[46,47]
Total phenolics (pulp)	16.4 ± 1.8 µg/mL	[46,47]
Total phenolic content (leaf)	12.01 ± 0.1 mg GAE/g	[50]
Total phenolic content (pulp)	16.4 ± 1.8 µg/mL	[46,47]
Total phenolic content (seed)	21.4 ± 1.4 µg TAE/mL ^e^	[46,47]
Total phenolic content (bark)	183.9 ± 5.6 to 619.4 ± 11.3 mg GAE/g	[45]

^a^ Values expressed as percentage leucocyanidin equivalents (LCE) per gram plant extracts; ^b^ Values expressed as rutin equivalent (RU) per gram of plant extracts. ^c^ Values expressed as catechin equivalents (CTE) per gram of plant extracts. ^d^ Values expressed as gallic acid equivalent (GAE) per gram of plant extracts. ^e^ Values expressed as tannic acid equivalents (TAE) per milliliter of plant extracts.
